# A Case of Migraine Treated Through Hybrid Consultation via In-Person and Online Telemedicine at an Occupational Health Nurse’s Office

**DOI:** 10.7759/cureus.71737

**Published:** 2024-10-17

**Authors:** Masahito Katsuki, Miho Ooka, Yasuhiro Wada, Yuki Nakata, Daiki Sato

**Affiliations:** 1 Physical Education and Health Center, Nagaoka University of Technology, Nagaoka, JPN; 2 Department of Neurosurgery, Tsubame-Sanjo Sugoro Neurospine Clinic, Sanjo, JPN; 3 Department of Electrical, Electronics and Information Engineering, Graduate School of Engineering, Nagaoka University of Technology, Nagaoka, JPN

**Keywords:** migraine, occupational doctor, occupational medicine, occupational nurse, online telemedicine, operational efficiency, remote medical care, school doctor, school medicine, school nurse

## Abstract

An occupational doctor cannot perform medical procedures, such as diagnosis and prescription. They can only give advice to the workplace. Online telemedicine facilitates workplace-doctor collaboration and may solve this problem. We present the first case of migraine treated by hybrid consultation via in-person and online telemedicine at the occupational health office. In the present case, a 36-year-old male had experienced headaches since age 15 and had been diagnosed with migraine. He was on prophylactic treatment with 10 mg of lomerizine, experiencing one monthly migraine attack, often relieved by 50 mg of sumatriptan. His Headache Impact Test-6 (HIT-6) score was 56 at the consultation. He visited the occupational health nurse’s office due to a migraine attack. An occupational doctor assessed him and diagnosed a migraine attack. The patient requested triptans, but only over-the-counter acetaminophen was available, and the doctor could not prescribe medication because the doctor was just an occupational doctor, and the nurse’s office was not a clinic under the Japanese Medical Act. The occupational doctor, who was also hired by the other clinic as a physician, conducted an online consultation via the clinic at the nurse’s office, diagnosed a migraine attack, and prescribed 50 mg of sumatriptan. The prescription was sent to a nearby pharmacy, and the patient found relief within 15 minutes after taking the triptan. Regular online consultation at the nurse’s office has been continued, and prophylactic medications were strengthened. His migraine frequency decreased once in five months, and the HIT-6 score improved to 50. Performing online telemedicine at the workplace, such as the occupational nurse’s office, could overturn the conventional wisdom that occupational physicians cannot perform medical treatment at non-medical institutions and can only refer patients to other clinics based on the Japanese Medical Act. Our case suggested the importance of strategic collaboration between occupational doctors and telemedicine-enabled medical facilities in ensuring seamless healthcare delivery, particularly for busy workers.

## Introduction

Since March 2020, the pandemic of the coronavirus disease 2019 (COVID-19) has significantly increased the demand for telemedicine to minimize the need for face-to-face consultations [1,2]. Until May 8, 2023, COVID-19 was classified as a Category 2 infectious disease in Japan. This classification required public health centers to carry out full surveillance, and substantial medical and operational resources were dedicated to tasks like hospitalization recommendations and work restrictions. Due to a shortage of hospital beds, patients with mild COVID-19 symptoms were advised to isolate at home or stay in hotels for care. The surge in fever patients and COVID-19 cases placed immense pressure on regular medical services, and hospitals faced bed shortages due to the number of COVID-19 admissions. Medical facilities were further constrained by the need to separate fever patients from other patients in the flow from entry to waiting rooms and exam rooms. In response, Japan officially lifted the ban on online medical services. Japan formally introduced online telemedicine for most medical conditions in April 2022 [3]. With this system, patients with fevers can be diagnosed and prescribed medication remotely, using over-the-counter test kits, without the need for a hospital visit. Similarly, patients with chronic conditions or other acute diseases can avoid unnecessary exposure to other patients by using online medical services.

Although telemedicine was initially implemented in response to the pandemic, it is now being adopted in specialized outpatient settings [4], such as headache clinics [5-7]. Online consultations for primary headaches are as safe and effective as in-person visits [8]. There is growing optimism that telemedicine, particularly video-based consultations, will be increasingly integrated with traditional in-person care. In online consultations, it is not necessary for the doctor to be in the same location as the patient, who can be at home, at work, or in the nurse’s office at workplaces or schools. The focus is now on how Japan can leverage the speed and convenience of telemedicine in the future.

An occupational doctor (or occupational health doctor) is a medical doctor specializing in occupational medicine, a field that focuses on workers' health, safety, and well-being. These doctors are trained to identify, prevent, and manage work-related injuries and illnesses, as well as to promote workplace health and safety practices. Key responsibilities of an occupational doctor include (1) preventing work-related health issues, i.e., identifying potential hazards in the workplace and developing strategies to reduce risks of illness and injury; (2) workplace assessments, i.e., conducting evaluations to determine if workplace conditions pose a risk to employees’ health, such as exposure to harmful chemicals, ergonomics, or stress; (3) medical surveillance, i.e., regularly monitoring the health of workers who are exposed to certain risks (e.g., toxic chemicals, loud noise); (4) rehabilitation and return to work, i.e., assisting employees in their recovery and facilitating a safe return to work after an injury or illness; (5) health promotion, i.e., implementing health and wellness programs to improve the overall well-being of employees, including mental health, fitness, and preventive care; (6) fitness for duty evaluations, i.e., assessing whether an employee is physically and mentally able to perform their job duties safely; and (7) regulatory compliance, i.e., ensuring that the workplace complies with legal and safety standards set by government or industry regulations.

Companies that employ more than 50 people in Japan must have a full-time industrial doctor. However, as the company is not a clinic as defined by the Japanese Medical Act, the doctors cannot perform medical procedures, such as diagnosis and prescription, and can only give advice to the workplace described above. The doctor can only write a referral letter to other hospitals if a patient needs treatment. The patients go there with the letters, which can be burdensome for busy workers. Moreover, in many small companies, there is not always an occupational physician. In some cases, the occupational physician only comes once a month, and in these cases, the occupational health nurse mainly provides health consultations.

In this context, we present the first case of migraine treated by hybrid consultation via in-person and online telemedicine at the occupational health office. Although industrial doctors are generally not able to prescribe medication in the company, they will now be able to issue prescriptions anywhere by online telemedicine, and this could be a way to help workers stay healthy.

## Case presentation

Twenty-one days before the initial consultation, a 36-year-old male was transferred to our workplace and had to move house. He has experienced headaches since he was 15 years old and was diagnosed with migraines by his previous doctor. He has been receiving prophylactic treatment with 10 mg of lomerizine for several years, experiencing one migraine attack per month. Lomerizine has been indicated for migraine prophylaxis in Japan since 1999 [9,10]. A dose of 50 mg of sumatriptan often provided relief. The Headache Impact Test-6 (HIT-6) score was 56 at his consultation.

One day, he visited the occupational health nurse’s office because of a migraine attack, rating the pain as four on a numerical rating scale. An occupational doctor was present, so he assessed the patient. The headache was described as pulsatile and unilateral, aggravated by physical activity. While there was no nausea, the patient reported phonophobia and photophobia. The occupational doctor diagnosed him with a migraine attack, and the patient requested a prescription for triptans. However, the only medication available at the nurse’s office was over-the-counter acetaminophen. In addition, the occupational doctor could not prescribe medication since the nurse's office was not a clinic but merely part of the workplace.

The nurse then informed the patient about online telemedicine covered by Japan's medical insurance system. The patient asked the doctor directly to perform an online consultation. The doctor went to another room, started his computer, and the patient used his smartphone. We then started the Digikar-Smart app (https://digikar-smart.jp/, M3 DigiKar, Inc., Tokyo, Japan) and performed through video consultation. The doctor, working part-time at another clinic, conducted the online consultation via the clinic, diagnosed the patient with a migraine attack, and prescribed 50 mg of sumatriptan for acute treatment. The doctor undertook the Chronic Headache Online Medical Treatment e-learning course that the Japan Headache Society offered and obtained its certificate. The prescription was faxed to a pharmacy near the patient's workplace (with the original sent by post), and the patient collected and took the triptan. The migraine attack was relieved within 15 minutes, avoiding further aggravation.

After that, the patient continued to receive monthly regular online consultations at the workplace nurse’s office and maintained prophylactic treatment with 10 mg of lomerizine. Due to hypertension noted at 140/92 mmHg, 20 mg of olmesartan (angiotensin II receptor blocker) was initiated 30 days after the initial consultation. Olmesartan may have a secondary migraine-prophylactic effect [11,12]. Over the past five months, the frequency of migraine attacks reduced to just once, and the HIT-6 score improved to 50 after 5 months. His blood pressure has been maintained around 120/70 mmHg.

**Figure 1:**
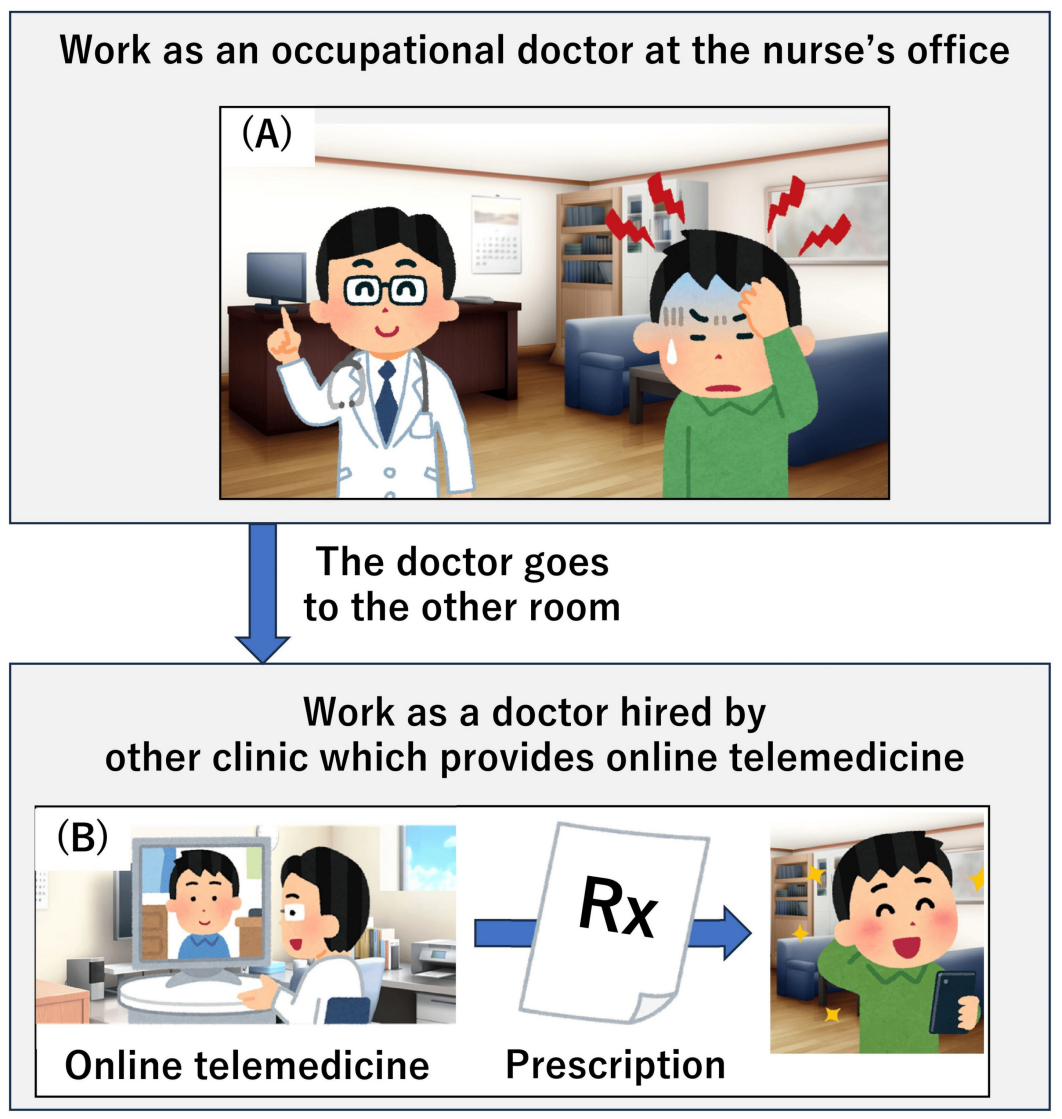
Schematic illustration of hybrid consultation via in-person and online telemedicine at the occupational health nurse’s office (A) Since the nurse’s office is part of the workplace, providing medical treatment or practice there is not allowed. In addition, occupational doctors cannot provide health insurance-covered medical care. (B) However, if an occupational physician is also employed by another clinic that offers online telemedicine, online medical care via that clinic can be conducted at the workplace. Thus, medical questionnaires, diagnoses, and prescriptions can be provided at the workplace through online telemedicine, resulting in efficient medical practice. Image credits: Masahito Katsuki, Irasutoya, Minchirie

The exchange, in this case, is illustrated in Figure 1.

## Discussion

We presented the first case of migraine treated by hybrid consultation via in-person and online telemedicine at the occupational health office. The patient's headache improved after being prescribed a triptan by online telemedicine under the health insurance system at a workplace that was supposed only to provide medical advice. Our practice can overturn the conventional wisdom that occupational physicians cannot perform medical treatment at non-medical institutions and can only refer patients to other clinics based on the Japanese Medical Act, leading to improving the busy worker’s health.

Changes in the online medical practice in Japan

In Japan, discussions about telemedicine began in 1997, but its use remained limited due to stringent regulations. In 2018, online medical care was approved for specific conditions, such as incurable diseases, though its adoption was minimal, with a utilization rate of just one in a million. By April 2020, restrictions were eased further, allowing online consultations for follow-up care of certain chronic diseases, in response to the strain on the healthcare system during the COVID-19 pandemic. Finally, in 2022, online medical care was approved for all conditions, including initial consultations. Initially, the main driver behind the expanded use of telemedicine was the need to reduce face-to-face contact to prevent the spread of infectious diseases and minimize in-person hospital visits. In recent years, however, the demand for online healthcare services has started to shift toward other uses.

One example is remote and rural medicine. While some remote areas have offered online medical care for the residents, its use has been limited, and many municipalities struggle to secure enough physicians. Traditionally, doctors made home visits, but a new model is emerging where nurses visit patients in person, and doctors provide care remotely from their clinics. A significant negative correlation (r = -0.31) between the availability of telemedicine and population density suggests that telemedicine is being used more in less populated areas to ensure access to medical care [13]. However, some patients, such as the old or those unfamiliar with technology, face challenges in using online medical services [14]. In response, municipalities and hospitals have begun deploying vehicles equipped with nurses and basic laboratory test equipment to visit patients' homes, where nurses assist them in using devices for online consultations with doctors. In addition, the prohibition on online care at community centers, schools, and other locations outside homes and hospitals is being lifted. To make online medical care more accessible, user-friendly devices for video consultations are being developed. With Japan facing a shortage of doctors and an aging population, online medical care could be crucial in providing equitable healthcare access across regions [15].

Another area of focus is the use of specialized outpatient clinics [16]. For instance, with fewer than 1,000 headache specialists in Japan, many patients cannot access a specialized headache clinic in person. For these patients, online medical services offer appropriate preventive treatment and guidance [3,5]. Online psychiatric care has also started (https://fastdoctor.jp/mental/) due to long waiting times for appointments. Psychiatrists from across Japan are sharing tasks, allowing patients to receive care from available doctors nationwide. Online telemedicine is also being used to prescribe and monitor obstructive sleep apnea treatment, particularly for long-term continuous positive airway pressure therapy [17]. Moreover, reports on the outcomes of online consultations for lifestyle-related diseases [18-20] and psychiatry [4] are gradually emerging. As a result, some medical institutions are now addressing unmet healthcare needs by sharing patients across the country, even when specialty outpatient clinics are not available nearby.

In addition to medical care in remote or rural areas and specialized outpatient services, we propose online medical care in collaboration with occupational doctors and school doctors. The main job of both occupational doctors and school doctors is to provide advice to workplaces and schools. Since workplaces and schools do not meet the medical clinic criteria, it is impossible to provide medical treatment under Japan's Medical Act. For this reason, even if someone needs treatment, the only thing occupational and school doctors can do is to write a referral letter, which is a bit of a letdown. In 2018, it was reported that 0.4% of employees at all workplaces in Japan were on leave due to mental illness [21]. The economic losses in Japan due to migraine and insomnia are over two trillion yen [22] and 18 trillion yen, respectively [23]. Other mental illnesses, depression, and social anxiety disorder also cause significant economic losses. However, many people do not visit the hospital because they are busy, which can sometimes lead to the progression of their condition. We propose a third new application of online medical care: providing online medical care in non-medical institutions such as workplaces and schools. If staff members feeling unwell are prescribed medication promptly, their condition will be improved while their symptoms are mild. Even though the economic benefits of treatment are clear, patients don't have time to go to the hospital. In such areas, the convenience of online medical consultations could be practical. We will accumulate clinical data on this medical practice and report on its effectiveness in the future.

Treatment needs for migraine attacks

Headaches are a widespread public health issue, with primary types such as migraines, tension-type headaches, and trigeminal autonomic cephalalgias classified in the International Classification of Headache Disorders. In Japan, migraine prevalence ranges from 0.9% to 9.5%, tension-type headaches from 15% to 20% of the population [24], and medication-overuse headaches 2.3% [25]. The Clinical Practice Guideline for Headache Disorders 2021 [9] outlines treatment strategies for migraine attacks. Acute treatment typically involves acetaminophen, non-steroidal anti-inflammatory drugs, triptans, and lasmiditan. For those with migraine attacks that occur more than twice a month, prophylactic treatment may include lomerizine, propranolol, valproic acid, amitriptyline, angiotensin-receptor blockers, and anti-calcitonin gene-related peptide monoclonal antibodies.

Our case was diagnosed as migraine attack after the in-person and afterward online consultation and improved with sumatriptan. As prophylactic medications, in addition to the lomerizine that had been administered, olmesartan was added due to the patient's hypertension. Further control of the migraine was achieved. Although almost three-quarters of people with migraine in Japan may have potential unmet needs for acute treatment of migraine, only 5.3% of migraine sufferers have used triptans [26]. Moreover, prophylactic medication had been never prescribed for about 90% of migraine patients [27]. Online medical consultations at the workplace could effectively improve access to headache treatment, including triptan and prophylactic medications. Our hybrid consultation can also solve these problems.

On the other hand, triptans are the only disease-specific medications available in Japan for the acute treatment of migraine. However, triptans are contraindicated in people with cardiovascular disease and should be used with caution [28]. Online consultations cannot detect side effects that can only be detected through laboratory tests, such as liver or kidney damage caused by prophylactic medications. In medical consultation at the workplace or online consultations, unlike a consultation at a hospital with abundant medical resources, there is a possibility that the history of the patient's illness may be missed if it is not carefully listened to, and caution is required regarding the risk of using triptans and prophylactic medications.

Previous practice in online telemedicine and occupational medicine

There are many examples of workplace health promotion and occupational health studies that use online tools. For example, there are health check-ups that combine online and offline methods [29], and there are also studies that use wearable sensors and smartphone apps to evaluate the work environment [30]. Young doctors are interested in using these next-generation tools in medicine [31], but there are no reports yet on combining occupational health and medical practice covered by the official health insurance system. In addition, these are tools for evaluation and health promotion. They do not directly lead to actual treatment.

Even if a health check-up reveals that you have a lifestyle-related disease, around 20% of people do not visit a medical institution [32]. Even if a condition such as migraine, which is not directly life-threatening, is pointed out by an occupational nurse or doctor, the likelihood of seeking medical attention may be very low. Considering that migraine is a progressive disease [33] and reduces productivity [34,35], people should be encouraged to visit a medical institution as soon as possible, but busy workers are likely to avoid medical consultations. Our method could be useful for busy workers to access health-insurance-covered treatment at the workplace and improve their health.

Limitations and considerations

As a limitation, online telemedicine in the workplace should be cautious of secondary headaches due to the absence of radiological examinations. It is important to recognize that online medical care is provided at the patient's request, and occupational doctors and nurses should not indiscriminately arrange consultations. In addition, patient engagement, adherence, and access to specialized in-person care are crucial factors. Different laws, regulations, and reimbursement models across regions and countries necessitate careful navigation. Finally, this report is a case report, and further research is expected to be conducted to determine how much medical efficiency can be improved and what the treatment effect is under the hybrid consultation via in-person and online telemedicine at occupational health nurse’s offices.

## Conclusions

We presented the first case of migraine treated by hybrid consultation via in-person and online telemedicine at the occupational health office. The patient's headache improved after being prescribed a triptan by online telemedicine under the health insurance system at a workplace that was supposed only to provide medical advice. Performing online telemedicine at the workplace, such as the occupational nurse’s office, could overturn the conventional wisdom that occupational physicians cannot perform medical treatment at non-medical institutions and can only refer patients to other clinics based on the Japanese Medical Act. Our case suggested the importance of strategic collaboration between occupational doctors and telemedicine-enabled medical facilities in ensuring seamless healthcare delivery, particularly for busy workers. In the future, a further consultation style can be performed at once for headache specialists and patients with occupational physicians and nurses.
